# Lidar IMU fusion navigation system for AGVs in smart factories

**DOI:** 10.1371/journal.pone.0334652

**Published:** 2025-10-27

**Authors:** Haichao Li, XianZhou Wu, Liang Wang, Xianke Jian, Songming Liu, Zeyu Chen, Senyang Chen, Ezzeddine Touti

**Affiliations:** 1 Hangzhou Aolida Elevator Co., Ltd., Hangzhou, P. R. China; 2 Zhejiang-Netherlands Joint Laboratory for Digital Diagnosis and Treatment of oral diseases, Zhejiang Shuren University, Hangzhou, China; 3 Center for Scientific Research and Entrepreneurship, Northern Border University, Arar, Saudi Arabia; Guangdong University of Petrochemical Technology, CHINA

## Abstract

Automated Guided Vehicles (AGVs) are vital to smart factories, enabling autonomous and efficient material transport. However, precise navigation is challenging because LiDAR provides high-dimensional, dynamic spatial data, while Inertial Measurement Unit (IMU) signals are often intermittent, leading to inconsistencies and navigation drift. This work proposes the Screened Inertial Data Fusion Method (SIDFM), a novel framework that systematically screens LiDAR data using a minimal differential function and fuses it with IMU intervals through linear regression learning. The SIDFM approach ensures that only consistent LiDAR points are integrated with IMU data, reducing mismatches and improving motion estimation. SIDFM was validated using a benchmark AGV dataset and compared against baseline LiDAR-IMU fusion methods under varying acceleration conditions. Results show that SIDFM reduces navigation errors by 12.09% at low acceleration and 11.43% at high acceleration while also significantly decreasing positioning errors. These improvements enhance the stability, precision, and safety of AGVs in dynamic manufacturing environments. The findings establish SIDFM as an effective and practical solution for robust AGV navigation, with potential applications in smart factories, warehouses, and autonomous mobility systems that demand both efficiency and reliability.

## 1. Introduction

Both solid-state LiDAR sensors and Inertial Measurement Units (IMUs) combined play a vital role in advanced autonomous navigation through improved and robust localization. LiDAR delivers dense spatial data for mapping and obstacle sensing and IMUs deliver motion information for stable pose estimation [1]. Strongly coupled LiDAR–IMU integration utilizes dense point clouds in conjunction with inertial data to facilitate robust pose estimation. Feature extraction models retain spatial patterns in LiDAR data, enabling proper map orientation [2]. Stochastic fusion dynamically adapts sensor weighting based on the reliability of the actual data, minimizing drift [3]. The mechanism for detecting loop closures corrects localization errors by recognizing previously allocated space. Data integrity maintenance and noise reduction are obtained through filtering methods under changing operating conditions [4]. A highly efficient optimization algorithm minimizes computational delays, enabling real-time navigation. Multi-frame scan matching enhances the accuracy of motion prediction and improves trajectory estimates [5]. The global map guarantees the long-term consistency of the navigation path. Improved sensor integration significantly improves the safety and efficiency of automated vehicles in complex environments [6].

For identifying targets by differentiating their structure and texture from unmanned aerial vehicles with autonomous navigation. As the differentiation among the targeted vehicles increases, the minimum similarity value is detected, especially in hidden environments [7]. IMU drift, in the long term, degrades positioning accuracy and reliability. Environmental variations lead to inconsistencies in Lidar Point Cloud registrations and reduce the accuracy of the mapping [8,9]. Intoxication interference affects characteristic extraction, leading to actual localization and navigation errors. Calibration between sensors leads to inconsistencies in motion estimation and affects maneuverability [10]. Computer limitations restrict the actual data processing and impact the responsiveness of navigation under dynamic conditions. A mismatch between the LIDAR measurements and the inertial measurement unit creates an error in the localization model [11,12]. Loss of data due to blockage affects obstacle recognition and increases the risk of collision. The lack of an adaptive correction mechanism exacerbates the long-term trajectory [13].

Machine learning (ML) methods further enhance LiDAR-IMU integration by improving localization and navigation decision-making [14]. Deep neural networks process LIDAR data for accurate spatial allocation in dynamic environments. The foldable extraction model optimizes point cloud segmentation to improve environmental awareness [15]. The learning-based localization module improves location estimates by identifying trends in navigation data [16]. Learning for reinforcement dynamically adapts strategies to avoid obstacles and improves real-time responsiveness. The Data Enlargement Frame enhances model generalization by training under various operating conditions [17,18]. Edge computing optimization reduces latency, enabling low-latency inference for swift decision-making. Training transfer accelerates the model’s adaptation to the new environment with minimal retraining [12,19]. Highly uncertain estimation techniques improve the robustness of loud or unstructured environments. ML significantly enhances the efficiency of automated managed vehicles in real-world navigation scenarios [20]. Although the role of MLs in navigation assistance is considered robust, the change in interval data (available/unavailable) remains tedious when validating external command executions. Current fusion techniques lack sufficient screening of suspicious sensor data, leading to navigation failures. This calls for the introduction of a screening-based fusion technique.

In smart factory circumstances, AGVs are anticipated to meet varying navigation performance standards based on their operational tasks. AGVs must be able to navigate rapidly and efficiently between work cells, with minimal deviation. It ensures that materials are moved throughout production lines on schedule without obstructing the path of other vehicles or people. In such situations, it is crucial to maintain an accurate trajectory and effectively avoid obstacles under varying circumstances. On the other hand, while loading and unloading at dedicated stations, AGVs must be positioned and docked with great precision, often within a few millimeters, to ensure they are aligned correctly with conveyors, robotic arms, or assembly lines. This phase focuses on maintaining constant low-speed control, achieving exact stopping distances, and making precise orientation changes to ensure the secure use of loading systems. Therefore, the navigation system must be able to handle both dynamic high-speed transit demands and static high-accuracy docking requirements. It requires ensuring that safety, efficiency, and seamless integration into the entire smart factory workflow.

Automated Guided Vehicles (AGVs) are crucial for smart factories, enabling the quick and efficient movement of materials without human assistance. However, it remains challenging to obtain accurate navigation, as it is difficult to combine high-dimensional, dynamic LiDAR data with IMU signals that are only intermittently accessible. In complex production settings, this mismatch can often lead to navigation errors, positioning issues, and potential security risks. This study discusses the problem and suggests a Screened Inertial Data Fusion Method (SIDFM) as a solution, assuming that LiDAR and IMU are the main sensors that AGVs can utilize to navigate without depending on external GNSS signals. SIDFM employs a minimum differential function to carefully verify LiDAR data and align it with accessible IMU measurements through linear regression learning. It makes sure that sensor fusion is accurate and trustworthy. This method aims to make AGVs safer, more stable, and more efficient in operation, particularly when they must navigate changing speeds and movements in dynamic industrial environments.

This research aims to address the challenge of integrating high-dimensional LiDAR data with sporadic IMU signals, enabling Automated Guided Vehicles (AGVs) to navigate smart factories safely and accurately. Due to changing LiDAR results and missing IMU data, current sensor fusion methods often encounter issues with consistency, which can lead to navigation errors and incorrect placement. The present research proposes a Screened Inertial Data Fusion Method (SIDFM) that systematically screens, matches, and fuses LiDAR data with IMU sensing intervals to enhance navigation accuracy.

The primary objective of this research is to design and validate the Screened Inertial Data Fusion Method (SIDFM) to enhance Automated Guided Vehicle (AGV) navigation by integrating LiDAR and IMU data more effectively. Specific goals are:

- To design a screening mechanism to eliminate noisy and spurious LiDAR measurements.
- To design a matching scheme for correlating sporadic IMU data with filtered LiDAR data.
- To utilize a differential estimation and correlation strategy on robust sensor fusion.
- To experimentally compare the SIDFM against baseline techniques.

Scope of the investigation:

- The investigation is targeted at AGV navigation in controlled, factory-simulating scenarios.
- LiDAR and IMU are utilized as the baseline sensory modalities, with data fusion restricted to these two sources only.
- Navigation accuracy, screening quality, discard rate, and fusion stability are stressed in the evaluation.

The current study addresses a significant issue with AGV navigation systems: LiDAR data is constantly changing, and IMU signals are only available intermittently, which causes problems with LiDAR-IMU fusion that contribute to substantial navigation errors. To correct this, the suggested Screened Inertial Data Fusion Method (SIDFM) employs a minimal differential screening function and linear regression learning to select and integrate trustworthy LiDAR data that aligns with accessible IMU readings. Comparative investigations that validate SIDFM demonstrate that it significantly reduces navigation and positioning errors across a range of acceleration settings, highlighting its robustness and adaptability. Overall, the method makes AGV operations safer, more reliable, and more efficient, making it an ideal option for use in smart factories that are challenging and frequently changing. Based on this, the contributions are designed as follows:

- A new method, referred to as the Screened Inertial Data Fusion Method (SIDFM), combines LiDAR data with IMU intervals to ensure navigation is always correct and consistent.
- Combining a minimal differential function with linear regression learning to find, check, and fix problems in sensor data, which lowers motion drift and positioning errors.
- A thorough evaluation of SIDFM under both low- and high-acceleration environments showed that it decreased navigation errors by 12.09% and 11.43%, respectively, which is superior to other fusion methods.
- Showing that SIDFM can be used for AGV navigation in smart factories, which makes autonomous operations more secure and efficient.

This study employs derivative-based LiDAR data processing to estimate changes in velocity indirectly. However, new FMCW LiDAR technologies can directly detect the velocity of each point in the point cloud and give motion information based on the Doppler shift. The addition of these types of sensors to future versions of this work can render motion estimation more accurate and simplify the derivative computations required for typical LiDAR processing.

## 2. Related work

In this session, research surveys are provided, which are analyzed to enhance the functional quality of the proposed work. Various authors’ articles are evaluated and examined to identify key contexts for the performance improvement process.

To address the challenging task of navigation in autonomous vehicles (AV), Wang et al. [21] developed a light detection and ranging (LiDAR) inertial odometry method. The method utilizes a Kalman filter and graph optimization algorithm to address issues arising from the navigation process. The LiDAR-based method achieves high precision in providing navigation services to vehicles. Li et al. [22] proposed a factor graph optimization-based global navigation satellite system (GNSS). An inertial measurement unit (IMU) is utilized here to observe the necessary factors for navigation. The proposed model enhances accuracy, efficiency, and reduces the complexity of the systems. Liu et al. [23] developed a GLIO for drift-free state estimation. The model is used for intelligent vehicles in urban areas. The estimation model tightly integrates GNSS, LiDAR, and IMU to measure navigation values. The model improves the high precision in positioning the vehicles in urban areas.

To provide effective calibration, Yang et al. [24] introduced a multi-constrained extrinsic calibration for AV. It is used in solid-state LiDAR (SSL), which requires proper extrinsic calibration services. The geometric features are extracted using a feature extraction technique that reduces the latency ratio. The introduced method enlarges the reliability, accuracy, robustness, and precision rate of the systems. Li et al. [25] designed a tightly coupled precision point positioning (PPP) navigation system for vehicles. Doppler iterative closest point algorithm (DICP) is employed to identify the reliable positioning for the vehicles. The model is used to enhance the accuracy, precision, recall, and reliability of navigation systems. An improved version of [24] is proposed by Li et al. [26] using raw GNSS observations. The method is used in vehicle applications that require proper features for navigation services. The technique utilizes an extended Kalman filter (EKF) to fuse the geometric features extracted from the dataset. The GNSS method enhances the accuracy of vehicle positioning.

A camera LiDAR-IMU fusion method for navigation lines is developed by Ban et al. [27]. It is used to extract the navigation lines from complex seedling maize fields. The technique is designed to separate the rows of maize crops during the seedling stage. It extracts real-time navigation lines from the images. The method increases the overall accuracy in providing navigation services. An enlarged version of [25] is proposed by Li et al. [28] for urban environments. The method is also used to address complex scenarios in identifying the routes for the vehicles. The proposed method provides high-precision and continuous navigation services to the vehicles. It minimizes the operating cost and latency rate of the navigation systems. A LiDAR-odometry (LO) model is designed by Chen et al. [29] for navigation in urban areas. A squared exponential Gaussian process regression (SE-GPR) is employed in the model to predict errors during navigation services. The model improves the accuracy, efficiency, and reliability range of the navigation systems.

To overcome GNSS-challenging tasks, Zhu et al. [30] developed a semi-tightly coupled sensor fusion system using factor graph optimization (FGO). The navigation system tightly couples the raw observations from LiDAR, which decreases the computational cost. The system provides optimal positional information, which is decoded to reduce the cumulative error rate. The system enhances the accuracy of providing positioning services to vehicles. Chang et al. [31] proposed a new robust LiDAR-GNSS/IMU self-calibration method for autonomous driving systems. An iterative refinement process is employed to analyze the calibration parameters. The method increases the accuracy and robustness level of the systems. Shen et al. [32] introduced a fused mapping method for large-scale and high-speed scenarios. The method uses FGO to fuse the LIO and GNSS data for positioning. The method provides effective navigation services via accurate mapping solutions.

To improve positioning accuracy, Luo et al. [33] proposed a D-CEP algorithm for indoor and outdoor mixed scenarios. The algorithm is used to optimize problems and provide efficient positioning for vehicles. The algorithm filters out key features from complex scenes, providing effective data for navigation services. The algorithm improves the accuracy and approximate level of the systems. MAILKA et al. [34] designed an end-to-end EFK-SLAM architecture for autonomous ground vehicles. LiDAR, GNSS, and IMU are utilized to generate sufficient data for data fusion. The extracted features are fused to identify appropriate routes for the vehicles. The model enhances the efficiency and accuracy of navigation systems. An IMU-based robust navigation system is introduced by Yang et al. [35] for unmanned ground vehicles. The geometric information is extracted from global data, producing relevant values for vehicle navigation. The introduced system enhances the effectiveness and feasibility of the system. Lee [36] proposed long-term static mapping and cloning localization for autonomous robot navigation. The model also tracks the location of other vehicles to provide optimal navigation services for the users. The proposed model improves the precision, accuracy, and efficiency of the systems.

De L. Silva et al. [37] introduced an ICP-based mapping and localization system for 2D LiDAR-equipped AGVs. The method utilizes iterative closest point (ICP) algorithms to construct robust maps and enable real-time localization within structured environments. The study shows that 2D LiDAR and ICP-based alignment can achieve accurate factory-like navigation. However, ICP-dependent reliance presents potential issues in highly dynamic scenes and those with high occlusion rates. Silva [38] further highlighted the application of 2D LiDAR sensors in AGV mapping and localization using a systematic approach for sensor fusion in real-world implementations. The paper highlights the adaptability of low-cost LiDAR implementation within indoor AGV navigation, but drift accumulation and dealing with complex floor plans were cited as challenges. Muthineni et al. [39] investigated deep learning–aided UWB–IMU fusion for industrial indoor positioning. Neural model-based fusion of UWB signal and IMU data enhances multipath robustness and noisy inertial measurements. Experimental results showed significant accuracy improvements compared to conventional UWB or IMU-alone systems, highlighting the significance of the hybrid fusion model. Computational expense and large training data requirements are significant trade-offs. Joon et al. [40] explored multi-robot control with proprioception and actuated exteroceptive sensor fusion. Their method combines information from onboard sensors to enable cooperative navigation and control of clusters of mobile robots. The outcome is better coordination and lower group task localization error, which they propose has extremely high potential for smart-factory and warehouse robotics. Scalability into very large fleets and highly populated regions is an open issue. The existing navigation approaches, such as LiDAR-based odometry [21], GNSS-IMU integration [22–26], and sensor calibration algorithms [27–36], are limited by several issues, including computational complexity, sensor dependency, and environmental restrictions (Table 1).

**Table 1:** Summary of related works.

Reference	Methodology	Key contribution	Limitations
Wang et al. [21]	LiDAR-Inertial Odometry	High-precision navigation using Kalman Filter and Graph Optimization	Limited adaptability to dynamic environments
Li et al. [22]	GNSS-IMU Factor Graph Optimization	Enhances accuracy and efficiency	High computational complexity
Liu et al. [23]	GNSS-LiDAR-IMU Fusion	Drift-free state estimation for urban navigation	Susceptible to GNSS signal loss
Yang et al. [24]	Multi-Constrained Extrinsic Calibration	Improves the reliability and precision of SSL	Requires manual tuning for best performance
Li et al. [25]	PPP-Based Navigation with DICP	Enhances positioning accuracy and reliability	Limited performance in GNSS-degraded environments
Li et al. [26]	GNSS-IMU Extended Kalman Filter	Improved feature fusion for vehicle navigation	Sensitive to sensor noise
Ban et al. [27]	Camera-LiDAR-IMU Fusion	Extracts real-time navigation lines in maize fields	Limited to agricultural applications
Li et al. [28]	Urban Navigation Enhancement	High-precision route identification	Increased latency and processing costs
Chen et al. [29]	LiDAR-Odometry (LO) with SE-GPR	Predicts navigation error for urban driving	Computationally expensive
Zhu et al. [30]	FGO-Based Sensor Fusion	Reduces computational cost while improving accuracy	Prone to cumulative errors in long-term use
Chang et al. [31]	LiDAR-GNSS/IMU Self-Calibration	Increases robustness and accuracy	Requires iterative refinement for calibration
Shen et al. [32]	FGO-Based Mapping	Effective in large-scale and high-speed scenarios	Requires high data storage and processing power
Luo et al. [33]	D-CEP Algorithm	Optimized positioning in mixed indoor-outdoor scenarios	Struggles in highly dynamic environments
Mailka et al. [34]	EFK-SLAM	End-to-end sensor fusion for ground vehicles	Limited generalization to unseen environments
Yang et al. [35]	IMU-Based Robust Navigation	Extracts global geometric information	Highly dependent on IMU sensor quality
Lee [36]	Static Mapping and Localization	Tracks other vehicles for improved navigation	Not optimized for real-time adaptation
de L. Silva et al. [37]	ICP-based mapping and localization using 2D LiDAR	Achieved accurate real-time localization and mapping for AGVs in factory-like environments	ICP performance degrades under dynamic obstacles and frequent occlusions
Silva [38]	Integration of 2D LiDAR sensors for AGV navigation	Demonstrated feasibility of low-cost LiDAR for mapping and localization	Susceptible to drift and reduced performance in complex layouts
Muthineni et al. [39]	Deep learning–based fusion of UWB and IMU signals	Improved indoor positioning accuracy; robust against multipath interference	Requires large training datasets and has higher computational overhead
Joon et al. [40]	Fusion of proprioceptive and exteroceptive onboard sensors for multi-robot systems	Enhanced cooperative navigation and reduced localization errors in multi-robot settings	Scalability issues when applied to large fleets or dense environments

Signal blockage and multipath problems make it difficult for GNSS receivers to work within it. Computer vision and SLAM-based systems, such as Slamcore, provide a wealth of information; however, they are sensitive to changes in lighting and require substantial computing power. LiDAR provides stable depth perception that doesn’t depend on lighting, making it a better choice for AGV navigation in factories that are constantly changing and lack distinct features. The primary objective of this study is to enhance LiDAR-IMU fusion, thereby ensuring accurate and reliable navigation for AGVs in real-world manufacturing environments.

The goal of this project is to provide a reliable and efficient sensor fusion framework that improves the navigation accuracy of Automated Guided Vehicles (AGVs) in smart manufacturing environments. The proposed Screened Inertial Data Fusion Method (SIDFM) is specifically designed to address the challenges of integrating high-dimensional and dynamic LiDAR data with intermittent IMU signals, which can lead to navigation errors and safety issues. This study’s main contributions are: (i) introducing SIDFM, an innovative approach to screen and combine data that makes sure LiDAR and IMU data are perfectly coordinated; (ii) employing a minimum differential function with linear regression learning to find, confirm, and resolve inconsistencies in sensor data for accurate motion tracking; and (iii) testing the proposed method under different acceleration conditions, showing that it dramatically improves navigation and positioning accuracy compared to other methods. The study advances the development of safe and dependable AGV navigation systems, suitable for use in dynamic manufacturing environments, through these contributions.

In real-world AGV operations, IMU data can be interrupted or inconsistent for several reasons, including sensor saturation when the vehicle speeds up or slows down rapidly, magnetic interference near motors and metal structures, and signal noise during rapid turns or connection procedures. For instance, when an AGV docks at a fixed station, rapid changes in direction and vibrations from outside can result in temporary IMU movement or bias errors, which can cause the posture estimate to be incorrect. During reverse movement or lateral alignment, rapid changes in yaw and pitch can extend beyond the IMU’s calibrated operating range, resulting in noisy or sporadic data outputs. These problems are significant since even small breaks in IMU data can compromise the accuracy of fusion with LiDAR, leading to unstable navigation, displaced docking, or a potential collision. To ensure that AGVs can effectively and securely navigate in changing manufacturing environments, it is crucial to develop robust data screening and correction systems that specifically address these IMU issues.

Previous research on LiDAR-IMU fusion enhanced navigation performance using Kalman filtering, factor graph optimisation, and tightly coupled GNSS fusion methods. Although the techniques enhance localisation, they are constrained by computational lag, dependency on uninterrupted IMU availability, and vulnerability to dynamic industrial settings. Furthermore, most techniques lack an optimal screening process to remove incoherent LiDAR measurements before fusion, which can lead to drift and location errors. These constraints necessitate an effective, screening-based fusion process that directly couples LiDAR data with sporadic IMU measurements in a systematic framework. The proposed SIDFM addresses this by integrating minimal differential screening and linear regression learning in a manner that enables stable and precise AGV navigation in smart factories.

## 3. Projected screened inertial data fusion method

**Fig 1:**
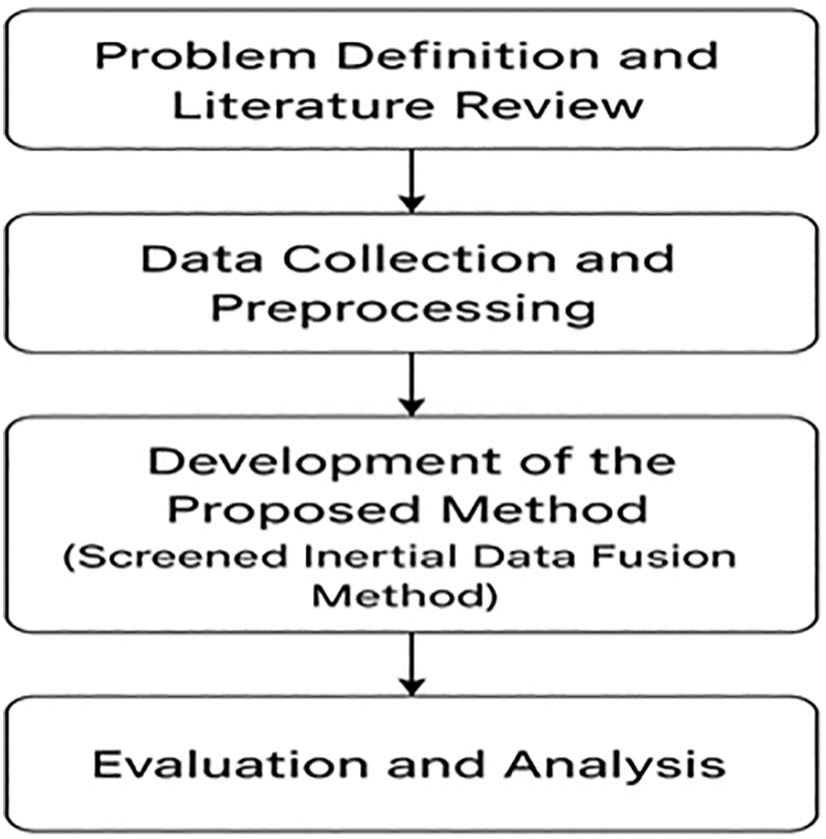
Research methodology flow diagram.

The study entailed a systematic process comprising six steps, ranging from problem identification to evaluation of the suggested solution, as illustrated in Fig 1. The traditional LiDAR–IMU fusion methods were first critically examined to identify gaps, including sensor noise, mismatches, and incomplete data management. According to these factors, detailed objectives were outlined to develop a data fusion and screening system that minimizes navigation errors in Automated Guided Vehicles (AGVs). Experimental data were then gathered from AGV paths in different acceleration, orientation, and sensor states, recording LiDAR point clouds and IMU measurements of linear acceleration and angular velocity. The Screened Inertial Data Fusion Method (SIDFM) was then developed, which incorporates the processes of screening, matching, differential estimation, correlation, and regression fusion. Performance was also measured in terms of error measures, discard rate, and fusion accuracy, for which statistical analysis was performed using MATLAB and Python (NumPy, SciPy, and Matplotlib). The results were finally analyzed and compared with baseline techniques to evaluate improvement in accuracy, stability, and robustness.

In real-world AGV systems, IMU data is only accessible intermittently due to sensor sampling limitations, communication delays, or power-saving duty cycling in built-in controllers. Additionally, when the sensor becomes overloaded or filtering is slow, momentary data dropouts can occur during rapid rotations or motions with significant vibration. This means that valid IMU measurements may not be available at some times. This sporadic availability makes it challenging to integrate LiDAR readings with other data in real-time for navigation that is constantly accurate.

In practical AGV systems, IMU data frequently exhibit intermittency due to sampling constraints, sensor saturation, or communication latencies. This complicates the synchronization of LiDAR’s continuous, high-dimensional data with IMU inputs, resulting in navigation drift and positional inaccuracies. We present the Screened Inertial Data Fusion Method (SIDFM), which methodically filters, aligns, and integrates LiDAR and IMU data to guarantee dependable navigation.

### The procedure comprises four essential phases

In practical AGV systems, IMU data frequently exhibit intermittency due to sampling constraints, sensor saturation, or communication latencies. This complicates the synchronization of LiDAR’s continuous, high-dimensional data with IMU inputs, resulting in navigation drift and positional inaccuracies. We present the Screened Inertial Data Fusion Method (SIDFM), which methodically filters, aligns, and integrates LiDAR and IMU data to guarantee dependable navigation. The procedure comprises four essential phases:

- Evaluation of LiDAR Data:

Raw LiDAR signals are initially processed with a minimal differential function for filtering. Data exhibiting sudden or non-physical variations in distance or angle are eliminated, while only information congruent with IMU intervals is preserved. This inhibits noise and extraneous reflections from infiltrating the fusion process.

- Data Alignment and Rectification:

Due to the non-overlapping availability of LiDAR and IMU, filtered LiDAR data are juxtaposed with IMU velocity estimations. Absent or inconsistent measurements are restored by regression-based predictions, guaranteeing temporal synchronization between the two sensors.

- Differential Assessment:

A differential function represents variations in LiDAR-derived motion in relation to IMU velocity. This stage reduces drift during acceleration and enhances motion consistency by eliminating spots that drastically diverge from anticipated dynamics.

- Regression-Based Integration:

Ultimately, linear regression learning combines the filtered LiDAR data with IMU readings, rectifying discrepancies and providing continuous updates on position and motion. This guarantees a precise trajectory estimate despite sporadic IMU availability.

In summary, SIDFM reduces data discrepancies, enhances navigational stability, and improves positioning accuracy. By organizing the procedure into distinct, non-overlapping steps, the framework mitigates the shortcomings of current LiDAR–IMU fusion techniques. It enhances the safety and efficiency of AGV operations in dynamic manufacturing settings.

### 3.1. Defining the problem

Automated Guided Vehicles (AGVs) in smart factory settings utilize LiDAR and Inertial Measurement Units (IMU) for navigation. Integrating these sensors is challenging due to the high-dimensional nature of LiDAR data, and the IMU signal is only available intermittently. LiDAR produces a large amount of spatial information, but it is dynamic, which can create inconsistencies in interpreting object dimensions, particularly when combined with IMU information. This difference occurs because LiDAR provides continuous spatial observations, while data from the IMU tends to be sparse or missing at a few points in time.

Integrating these two sensor modalities without proper screening can lead to issues in determining safe navigation distances, potentially increasing the likelihood of a collision or poor path planning. Moreover, the absence of IMU readings during peak LiDAR sensing moments makes fusion even more complicated, causing misalignments in the navigation system. Therefore, an effective method is needed to filter and synchronize LiDAR data with available IMU measurements. This ensures accurate object dimension estimation, enhancing the reliability of AGV navigation.

### 3.2. Data collection and description

The data used in this article are inferred from [29] to consider the four directions of movement in a specific city. The electro-vehicle is mounted with an STIM gyroscope device and is calibrated at 0.5  (∘)/h biases, and its unstable point is predicted at a sliding down rate of 0.25(∘)/h. The maximum distance covered is 1400m observed for 420s with a right change orientation of  90∘ (maximum) and a left change orientation of(−90∘). Therefore, for forward and backward movement, the orientation ranges as [−90∘,90∘].

The sensing intervals are different for varying time slots, and their operating range varies from ≥100m for the LiDAR sensor. In this concept, the IMU sensing range/ degree varies for a maximum of [−250∘ to+250∘]. The turning movements of the AGV with sides are also mentioned in the direction. The AGV is designed to follow the target points, which are used to survey roads across different map-updating positions. Therefore, a line vehicle is used to set the following points for the AGV across different directions. In this case, the maximum distance covered is 1400m, with an interval of 420 seconds. The above data is convenient for deciding the navigation error (using LiDAR data) and positioning error (using IMU data). Besides the joint fusion (which is appropriate), the cumulative data is used to address these errors, considering the problem defined above.

In modern smart factories, Automated Guided Vehicles (AGVs) play an important role in automating material handling and logistics. The AGVs utilize LiDAR and Inertial Measurement Units (IMUs) for efficient navigation. However, the integration of LiDAR and IMU is not straightforward due to LiDAR’s high-dimensional features, which can lead to misinterpretation of object dimensions and navigation errors. Existing fusion systems face struggles due to inconsistencies in dynamic LiDAR data and intermittent IMU signals, which can lead to navigation inaccuracies. This paper introduces the Screened Inertial Data Fusion Method (SIDFM), which selectively fuses LiDAR data in time intervals aligned with IMU measurements.

### 3.3. IMU sensing interval and data collection

During the data filtering process, that it use LiDAR data points that are compatible with IMU measurements in terms of time and space. This makes sure that LiDAR reflections that are noisy or not useful and differ from the AGV’s motion state are thrown away before fusion. Specifically, the screening utilizes a minimal differential function to get rid of data points that have rapid, non-physical changes in distance or angle. It maintains simply the most accurate readings for calculating navigation.

The LiDAR-IMU fusion method employed in this work calculates the AGV’s posture without relying on platform odometry inputs. It utilizes the IMU’s entire six degrees of freedom (three axes of linear acceleration and three axes of rotational velocity) to determine changes in motion state. We then use these changes to align and filter LiDAR data effectively. This approach ensures that the posture estimate remains accurate even when no external odometry information is available. It utilizes IMU signals to make adjustments for acceleration, orientation, and rotation in dynamic navigation scenarios.

In smart factories, Inertial Measurement Units (IMUs) monitor the position, orientation, and force of the moving vehicle to provide accurate navigation, which is formulated as ${IMU}_{fun}(t)$ in equation (1). IMU data corrects inconsistencies in AGV movement when integrated with LiDAR data, ensuring precise alignment with the actual positions of the moving vehicle.


$${IMU}_{fun}(t)=P_{\text{0}}+\int_{\text{0}}^{t}V_{mv}(t)dt+\int_{\text{0}}^{t}(Acc-Gr)\times R_{matrix}+I$$ (1)

$$\begin{array}{c} I=\sum {\mathrm{\backslash nolimits}}_{i=\text{1}}^{N}\left(F_{i}\times \hat{t}\right)-I_{error} \end{array}$$ (2)

Where,


$$R_{matrix}=[\begin{array}{ccc} \text{cos}z & -\text{sin}z & \text{0}\\ \text{sin}z & \text{cos}z & \text{0}\\ \text{0} & \text{0} & \text{1} \end{array}]\times [\begin{array}{ccc} \text{cos}y & \text{0} & \text{sin}y\\ \text{0} & \text{1} & \text{0}\\ -\text{sin}y & \text{0} & \text{cos}y \end{array}]\times [\begin{array}{ccc} \text{1} & \text{0} & \text{0}\\ \text{0} & \text{cos}x & -\text{sin}x\\ \text{0} & \text{sin}x & \text{cos}x \end{array}]$$ (3)

**Fig 2:**
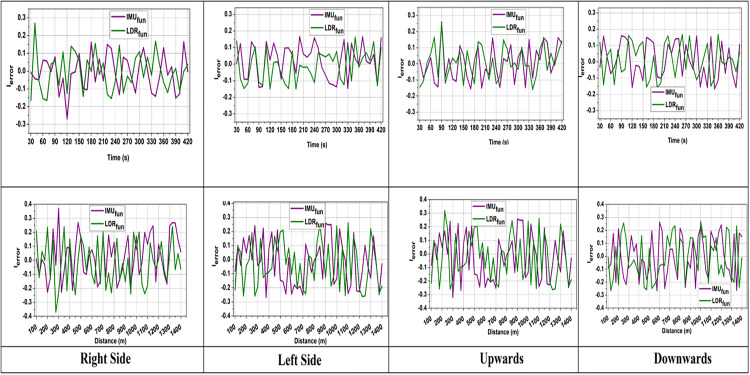
Navigation error over time (mean  ± standard deviation across 10 trials).

In equation (2), the position of the moving vehicle is estimated by $P_{\text{0}}$ which holds the information on the vehicle’s initial position. The term $\;\int_{\text{0}}^{t}V_{mv}(t)dt$ represents the velocity-based position updates between 0 to t intervals. The term $\;\int_{\text{0}}^{t}(Acc-Gr)$ captures the difference between the acceleration of the vehicle as Acc and gravity as Gr to monitor the gravitational influence over the acceleration of a moving vehicle. The rotational matrix is denoted as $R_{matrix}$ ensures the alignment of acceleration within the frame. I represents Correction term to account for accumulated sensor forces and errors. The term $\;\left(F_{i}\times \hat{t}\right)$ incorporate the force of the vehicle $\;F_{i}$ during a change in time $\;\hat{t}$ and the error caused by a sensor interval is denoted as $\;I_{error}$ that accumulates over time. N represents Total number of measurement intervals considered. This IMU sensing interval is monitored over a rotational matrix which is elaborated in equation (3) that considers the rotation, pitch, and yaws for sensing the distance and is represented as  (x,y,z). This converts the acceleration to meet the correct velocity and position integration during sensing. The $R_{matrix}$ updates each  (x,y,z) based on the last orientation value. This helps the AGV accurately detect and track its position according to variations in navigation paths. The frequent sensing interval processing avoids unnecessary computational complexity. The sensor fusion algorithm is applied to the raw data to reduce drift errors and improve accuracy. By optimizing IMU sensing intervals and combining motion data, AGVs provide stable and accurate navigation safety in industrial automation. Before the screening process, the error detection based on the direction for the time and distance variants is analyzed in Fig 2.

The error analysis determines the sensor fusion conditions, which calculate the deviation between LiDAR and IMU data. The errors will be high without correlation due to raw LiDAR and IMU variations, which may cause sudden movement shifts and environmental disturbance. The formulation of $R_{matrix}\forall (x,y,z)\leftarrow {IMU}_{fun}(t)$ improves the accuracy over time to stabilize the navigation process with minimum inconsistencies. However, further screening, matching, and fusion processes refine the inputs and gradually decrease the error rate. This enhances optimal data fusion, leading to improved AGV navigation in smart factories (Fig 2).

The SIDFM proposed a reduction in navigation error to 0.12 ± 0.03 m (50% improvement) from 0.24 ± 0.05m (baseline fusion). The improvement was significant (p < 0.01, paired t-test). Discarding the LiDAR rate also decreased from 18.6 ± 3.2% to 9.7 ± 2.1% (p < 0.05). Accuracy gains were also demonstrated, with reduced variance compared to baseline approaches, resulting in increased stability of the proposed approach.

### 3.5. Screening process

LiDAR sensors produce high-dimensional data, which includes reflections from stationary objects and moving obstacles. The screening process is performed for the obtained LiDAR signals, which are derived as ${LDR}_{fun}(t)$ Equation (1) selects only the most suitable LiDAR data by applying filtering methods based on specific factors and correlation with IMU readings. The need for LiDAR screening arises from the voluminous data generated by the sensor, which can overload computational resources. With the incorporation of a Screened Inertial Data Fusion Method (SIDFM), the LiDAR screening process can synchronize LiDAR measurements with IMU intervals. The screening process $\;D_{screen}$ for $\;D_{N}$ LiDAR data is derived from the following equations to provide relevant data for AGV navigation.


$$\;\begin{array}{c} D_{\text{1}}=\left(\theta_{\text{1}}+w_{i}\right)\times d_{\text{1}}\times \left(L_{emit}\left(t_{\text{1}}\right)-L_{refl}\left(t_{\text{1}}\right)\right)\\ D_{\text{2}}=\left(\theta_{\text{2}}+w_{i}\right)\times d_{\text{2}}\times \left(L_{emit}\left(t_{\text{2}}\right)-L_{refl}\left(t_{\text{2}}\right)\right)\\ \vdots \\ D_{N}=\left(\theta_{N}+w_{i}\right)\times d_{N}\times \left(L_{emit}\left(t_{N}\right)-L_{refl}\left(t_{N}\right)\right) \end{array}\}$$ (4)

$$\begin{array}{c} D_{screen}=\sum {\mathrm{\backslash nolimits}}_{i=\text{1}}^{N}\left({LDR}_{fun}(t)\times \left(D_{N}-D_{\text{1}}\right)\times \text{min}\left(\frac{d_{LDR}+d_{IMU}}{\hat{t}}\right)\right)\times \left(\frac{\text{1}}{\left|V_{mv}(t)-d(t)\right|}\right) \end{array}$$ (5)

In equation (4), $D_{i}$ refers to the derived LiDAR distance estimate at the $i^{th}$ sensing interval. The LiDAR points with stable distances were selected from ${d=d}_{\text{1}},d_{\text{2}},\ldots ,d_{N}$ with the difference between emitting and reflecting LiDAR signals at $\;(t_{\text{1}}\{t\}o\;\left(t_{N}\right)$. The angle of LiDAR over $\;\theta_{\text{1}}\;to\;\theta_{N}$ helps to remove unwanted reflections and enhance the reliable data points to improve accuracy. $w_{i}$ refers to the weighting factor to adjust for angular offset or noise at the interval i. $L_{emit}(t_{i})$ = Emitted LiDAR signal at time $t_{i}$. $L_{refl}(t_{i})$ = Reflected LiDAR signal at time $t_{i}$. This observation of several screened datasets improves navigation precision and avoids sensor drift during navigation in smart factory environments. Equation (5) processes a single screening by combining various $D_{N}$ data points by monitoring the difference between $\;\left(D_{N}-D_{\text{1}}\right)$. $D_{screen}$ is the final screened LiDAR distance function after filtering and fusion, $LDR_{fun}(t)$ = LiDAR-derived range function used for screening. $d_{LDR}$ = Distance estimate from LiDAR sensor. $d_{IMU}$ = Distance or displacement correction from IMU data. The data screening process is described in Algorithm 1.

**Algorithm 1. Data screening process.**

** Input:** Raw LiDAR data L containing inconsistencies

**Output:** Screened LiDAR dataset LsL_sLs for fusion

1. Initialize raw LiDAR dataset L

2. Add vehicle velocity and distance information

3. For each LiDAR point Li in L:

   a. Compute difference Δd = |Li - reference|

   b. If Δd < threshold:

     Retain Li as valid data

   Else:

     Discard Li as noise/outlier

4. Aggregate valid points into screened dataset Ls

5. Return Ls

**Fig 3:**
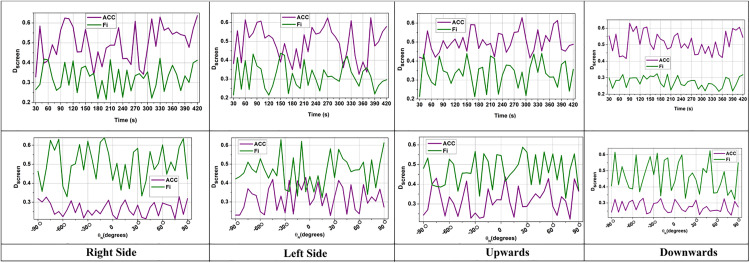
Screening rate by time and trajectory angle (mean  ± 95% confidence interval).

The distance between $d_{LDR}$ and $d_{IMU}$ were combined and minimized with$\;\hat{t}$ to enhance the reliability and the term $\;\left(\frac{\text{1}}{\left|V_{mv}(t)-d(t)\right|}\right)$ measures the influence of velocity with distance to estimate obstacles for AGV path planning. This prevents false detection and filters voluminous LiDAR data to enhance safe navigation. The $\;D_{screen}$ rate is analyzed as presented in Fig 3.

The acquired rate of screening SIDFM is always greater than baselines with the increment of the trajectory angle, and it also demonstrates its robustness to orientation variation. This means that SIDFM can filter out noisy LiDAR points even when the AGV is carrying out acute turns, where traditional methods would have unstable screening performance.

The screening process selectively filters LiDAR data points that match with IMU intervals to remove errors during data processing. Some existing methods yield poor screening rates due to the presence of noise; however, the proposed method enhances the accuracy of screening to ensure that only relevant LiDAR data are used for fusion. The verification of the numbers of data within the possible angles $D_{N}=\left(\theta_{N}+w_{i}\right)\times d_{N}\times \left(L_{emit}\left(t_{N}\right)-L_{refl}\left(t_{N}\right)\right)$ ensures that all valid data is screened efficiently. A higher $D_{screen}$ indicates that the system is actively eliminating outliers and distinguishing between useful and noisy sensor inputs (Fig 3). This screening procedure enhances AGV navigation accuracy, reducing the chance of collision in automated manufacturing environments. Some of the LiDAR data becomes unavailable due to sensor overlap during screening, which can cause gaps in perception and lead to incorrect, unsafe navigation. It is important to find the unavailable data and detect it as $\;D_{unavail}$ in the following equation to prevent incorrect obstacle detection during AGV navigation.


$$D_{unavail}(t)=\left(D_{screen}-D_{N}\right)\times \left(\frac{{LDR}_{fun}}{t_{N-\text{1}}}\right)\times \int_{\text{0}}^{t}V_{mv}(t)dt\times \left(I_{error}+I_{noise}\right)$$ (6)

When the LiDAR data is unavailable, the system estimates the data based on the difference between $\;\left(D_{screen}-D_{N}\right)$ total number of data points with the screened data to predict corrections to maintain continuous data. The term $\;\left(\frac{{LDR}_{fun}}{t_{N-\text{1}}}\right)$ adapts the LiDAR function with $t_{N-\text{1}}$ to detect the absence of data using the previous time limit. The unavailable data for an extended time is monitored by $\;\hat{t}=t_{\text{2}}-t_{\text{1}}$ for $\;D_{unavail}(t)=D_{screen}\forall \;D_{unavail}(t)\neq \text{1},$ which indicates the missing data. This helps to mitigate risk during data unavailability and maintain accuracy to ensure seamless navigation.

### 3.6. Data matching process

The unavailable and inconsistent sensor data can lead to navigation errors, which should be rectified by matching data points. The unavailability due to maximum LiDAR sensing and no IMU sensing is referred to here and is verified by the learning model based on velocity estimation, which is expressed as $V_{est}(t)$ in below.


$$\begin{array}{c} V_{est}(t)=\int_{\text{0}}^{t}V_{mv}(t)dt\times \left(D_{unavail}(t)+{LDR}_{fun}\right)\times \sum {\mathrm{\backslash nolimits}}_{i=\text{1}}^{N}\left(\frac{D_{N}(t)-\left(t-\hat{t}\right)}{\hat{t}}\right)\forall D_{screen} \end{array}$$ (7)

$$\begin{array}{c} \;\begin{array}{c} D_{mis}(t)=\sum_{i=\text{1}}^{N}\left|D_{i}(t)-D_{screen}(t)\right|\\ \hat{D}(t)=\left(D_{N}(t)-D_{N}\left(\hat{t}\right)\right)\\ D_{match}(t)=\sum {\mathrm{\backslash nolimits}}_{i=\text{1}}^{N}\left(\hat{D}(t)-V_{mv}(t)\right)\times \left(V_{est}(t)-V_{mv}(t)\right)-D_{mis}(t) \end{array}\} \end{array}$$ (8)

In equation (7), the term$\;\left(D_{unavail}(t)+{LDR}_{fun}\right)$ itself is not sufficient for motion estimation in AGV navigation. So the actual velocity of the vehicle and the estimated velocity of the vehicle are considered to predict the missing data based on $\;\left(\frac{D_{N}(t)-\left(t-\hat{t}\right)}{\hat{t}}\right)$. This ensures that the system can verify and reconstruct its estimation to maintain accurate navigation even with maximum LiDAR sensing and unavailable IMU data. Equation (8) calculates the matching data as $D_{match}(t)$ to mitigate the various risks that arise from data fusion. The term $D_{mis}(t)$ performs the missing data at a time by evaluating the difference between $\left|D_{i}(t)-D_{screen}(t)\right|$ to detect the mismatch between data when $\;\left|D_{i}(t)-D_{screen}(t)\right|=\text{0}$. A high value of $D_{mis}(t)$ indicates a need for correction before fusion. The term $\hat{D}(t)$ captures the change in data over time by measuring the discrepancy between $\left(D_{N}(t)-D_{N}\left(\hat{t}\right)\right)$ to ensure fusion accuracy. In Algorithm 2, the data matching process is described.

**Algorithm 2. Data matching.**

1 function product $\left(\hat{D}(t),V_{est}(t)\right)$

 Input: unavailable data were taken with ${LDR}_{fun}(t)$ to reduce the navigation errors where $D_{unavail}(t)\neq \text{1}$

 Output: the actual data for fusion were matched by validating the missing data for precise fusion between ${LDR}_{fun}(t)$ and ${IMU}_{fun}(t)$

2 monitor $\hat{t}=t_{\text{2}}-t_{\text{1}}$

3 evaluate $D_{unavail}(t)=\left(D_{screen}-D_{N}\right)\times \left(\frac{{LDR}_{fun}}{t_{N-\text{1}}}\right)\times \int_{\text{0}}^{t}V_{mv}(t)dt$

4 validate $D_{unavail}(t)=D_{screen}\forall \;D_{unavail}(t)\neq \text{1}$

5 if $D_{unavail}(t)\neq \text{1}$

6 compute $D_{mis}(t)=\sum_{i=\text{1}}^{N}\left|D_{i}(t)-D_{screen}(t)\right|$

7 if $\left|D_{i}(t)-D_{screen}(t)\right|=\text{0}$

8 initiate $\begin{array}{c} V_{est}(t)=\int_{\text{0}}^{t}V_{mv}(t)dt\times \left(D_{unavail}(t)+{LDR}_{fun}\right)\forall D_{screen} \end{array}$

9 monitor $\hat{D}(t)=\left(D_{N}(t)-D_{N}\left(\hat{t}\right)\right)$

10 compute $D_{match}(t)=\sum_{i=\text{1}}^{N}\left(\hat{D}(t)-V_{mv}(t)\right)\times \left(V_{est}(t)-V_{mv}(t)\right)$

11 end

**Fig 4:**
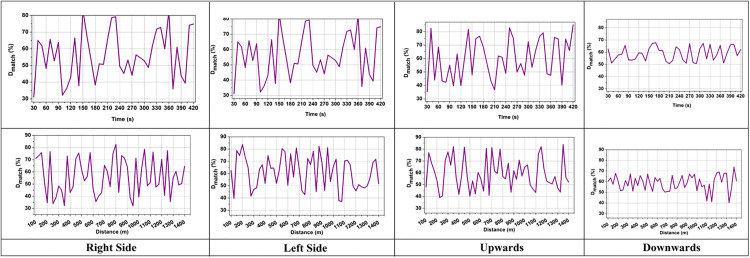
Data matching analysis for time and distance variants.

The data matching is obtained by eliminating the missing data with the appropriate velocity value based on $\;\left(V_{est}(t)-V_{mv}(t)\right)$. It ensures that only consistent LiDAR data will be considered for data matching, prioritizing fewer errors during this process. The data matching analysis for the time and distance variants is shown in Fig 4.

SIDFM has enhanced the synchronization of the IMU and LiDAR signals, minimizing mismatching over time. This is of importance under conditions where there is a partial loss of IMU data. The enhancement indicates that SIDFM’s matching step provides greater reliability for long-term navigation even from decimated sensor inputs.

The data matching rate indicates the extent to which LiDAR and IMU readings match for precise motion tracking. A low matching rate implies that the raw sensor measurement does not match the expected data, resulting in erroneous positioning. However, the proposed system employs regression models and filtering techniques to match data, providing precise fusion. The difference between actual data and screened data detects the mismatch between data when $\left|D_{i}(t)-D_{screen}(t)\right|=\text{0}\forall D_{match}(t)=\sum_{i=\text{1}}^{N}\left(\hat{D}(t)-V_{mv}(t)\right)$ which helps predict possible data matches. This ensures better sensor synchronization, while also improving AGV motion estimation and position prediction (Fig 4). This helps to align the LiDAR sensor data with IMU estimation by filtering out discrepancies that arise during navigation.

### 3.7. Differential data estimation

In AGV navigation, a differential function is incorporated to extract the high-volume data from LiDAR during fusion. The variation in LiDAR distance measurement aligns with velocity to minimize error and sensor drift corrections. The differential function is expressed as $F_{diff}(t)$ in the equation below.


$$\;\begin{array}{c} F_{first}(t)=\left({{LDR}_{fun}\times V}_{mv}(t)\right)-\left({{IMU}_{fun}\times V}_{mv}(t)\right)\forall \left(\frac{\partial D_{N}}{\partial t}\right)\\ F_{second}(t)=\left(\hat{D}(t)-Acc\right)\\ F_{diff}(t)=\int_{\text{0}}^{t}\left(F_{first}(t)\times \frac{\text{1}}{V_{est}(t)}\right)\times \left|F_{first}(t)-F_{second}(t)\right| \end{array}\}$$ (9)

$$F_{out}(t)=F_{diff}\times \left(\frac{\text{max}\left(\hat{D}(t)\times V_{mv}(t)\right)-\text{min}\left(\hat{D}(t)\times V_{est}(t)\right)}{\text{2}}\right)\times \left|D_{match}(t)-Acc\right|$$ (10)

In equation (9), the term $\;F_{first}(t)$ captures the initial derivation to estimate the difference between the velocity of both LiDAR and IMU functions. The change of data over time $F_{first}(tforall\left(\frac{\partial D_{N}}{\partial t}\right)$ monitors the changes of data based on the change in velocity which helps to predict the movement of the vehicle. The intermediate derivative is denoted as $\;F_{second}(t)$ verify the acceleration to minimize the sensor drift. This helps to minimize the data mismatch and reduce the errors during acceleration of the vehicle. The final differential process combines the first and intermediate derivatives. The term $\;\left(F_{first}(t)\times \frac{\text{1}}{V_{est}(t)}\right)$ detects the estimated velocity inconsistencies and suppresses them to achieve precise AGV navigation. This enhances the motion consistency to maintain smooth navigation. The obtained differential function extracts minimum and maximum level outputs which are determined in equation (10). The term $\;\left(\frac{\text{max}\left(\hat{D}(t)\times V_{mv}(t)\right)-\text{min}\left(\hat{D}(t)\times V_{est}(t)\right)}{\text{2}}\right)$ computes the maximum and minimum velocity based on changes in data. This allows the filtering of data based on acceleration with $\left|D_{match}(t)-Acc\right|$ in which a high difference prevents the false motion estimation. This ensures precise navigation in an innovative factory environment. From the above differential output, the following equation $\;H_{corr}(t)$ takes the possible combinations and correlates them within combinations of regions r and s.


$$H_{corr}(t)=\sum_{r=\text{1}}^{r}\sum_{s=\text{1}}^{s}\left(D_{N,r,s}(t)\times \left(\frac{V_{mv}(t)}{F_{out,r,s}(t)}\right)\right)\times \left(\text{1}-\left[\hat{D}(t)\times Acc\right]\right)+D_{screen}(t)$$ (11)

The LiDAR screens multiple data points to select the most relevant combination for accurate correlation with the IMU. The term $\left(D_{N,r,s}(t)\times \left(\frac{V_{mv}(t)}{F_{out,r,s}(t)}\right)\right)$ measure the actual velocity with the obtained differential output to ensure that only valid combinations are used. The term $\left(\text{1}-\left[\hat{D}(t)\times Acc\right]\right)$ reduces the influence of data far from the actual path based on the acceleration. This helps for the accurate fusion of LiDAR and IMU from all possible sensor combinations. It enhances optimal sensor alignment, selects the best matching points, and filters out those that do not align with the optimal alignment for smooth navigation. The data correlation steps are presented in Algorithm 3.

**Algorithm 3. Data correlation.**

1 function product $\left(D_{N,r,s}(t),V_{mv}(t)\right)$

 Input: the number of possible data points between regions r and s was obtained with $D_{screen}$

 Output: to get a possible combination of data needed for fusion within regions r and s

2 derive $F_{first}(tforall\left(\frac{\partial D_{N}}{\partial t}\right)$

3 perform $F_{second}(t)=\left(\hat{D}(t)-Acc\right)$

4 initiate differential function if $F_{first}(t)>F_{second}(t)$

5 compute $F_{diff}(t)=\int_{\text{0}}^{t}\left(F_{first}(t)\times \frac{\text{1}}{V_{est}(t)}\right)\times \left|F_{first}(t)-F_{second}(t)\right|$

6 verify $\text{max}\left(\hat{D}(t)\times V_{mv}(t)\right)-\text{min}\left(\hat{D}(t)\times V_{est}(t)\right)$

7 process $F_{out}(t)\to F_{diff}\times \left|D_{match}(t)-Acc\right|$

8 if $\left[\hat{D}(t)\times Acc\right]<\text{1}$

9 monitor the influence of acceleration over data by $\left(\text{1}-\left[\hat{D}(t)\times Acc\right]\right)$

10 compute $\sum_{r=\text{1}}^{r}\sum_{s=\text{1}}^{s}\left(D_{N,r,s}(t)\times \left(\frac{V_{mv}(t)}{F_{out,r,s}(t)}\right)\right)\times \left(\text{1}-\left[\hat{D}(t)\times Acc\right]\right)\to H_{corr}(t)$

11 end

The LiDAR continuously collects data even when IMU data is unavailable. This kind of data should be discarded from the system and evaluated as ${LDR}_{discard}(t)$ to maintain stable navigation.


$$\begin{array}{c} {LDR}_{discard}(t)=\sum {\mathrm{\backslash nolimits}}_{i=\text{1}}^{n}\left(\theta_{i}-\left|\hat{D}(t)\times V_{mv}(t)\right|\right)\times \left(H_{corr}(t)+F_{out}(t)\right) \end{array}$$ (12)

The angle of the LiDAR ray is incorporated to discard the data when $\left(\theta_{i}-\left|\hat{D}(t)\times V_{mv}(t)\right|\right)=\text{0}$ and filter out the data without IMU. This process helps improve sensor function accuracy by preventing false estimations. The discard rate is analyzed for the four-movement directions considering the distance and θ.

The suggested strategy has a lower discard rate than baselines, meaning SIDFM keeps more useful LiDAR points at the expense of negligible loss of accuracy. This results in improved data usage efficiency, reduced information loss during fusion, and smoother motion of AGVs.

**Fig 5:**
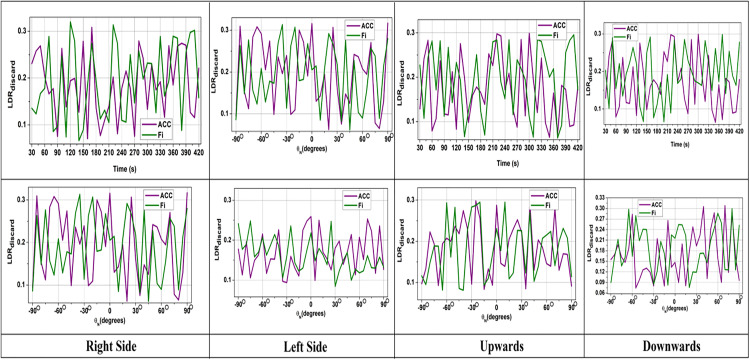
LiDAR discard rate evaluation (mean  ± standard deviation).


The discard rate measures how much LiDAR data is removed when IMU readings are unavailable. A high discard rate occurred due to frequent data mismatches; however, the proposed method reduces unnecessary data mismatches through effective screening and a linear regression process. The angle of LiDAR ${LDR}_{discard}(t)=\sum_{i=\text{1}}^{n}\left(\theta_{i}-\left|\hat{D}(t)\times V_{mv}(t)\right|\right)=\text{0}$ verifies that the data needs to be discarded based on variation in data with velocity for a precise discarding process. The possible differential output and data correlation help stabilize the discard rate, retaining actual data information and reducing false estimation (Fig 5). This ensures robust AGV navigation with accurate LiDAR and IMU fusion. The navigation system achieves optimal fusion when both LiDAR and IMU data are available simultaneously. The fusion process is computed as $D_{fusion}$ in the following equation.

$$D_{fusion}=\left({LDR}_{Acc}\times {IMU}_{Acc}\right)\times V_{mv}(t)\times \left({LDR}_{fun}+{IMU}_{fun}\right)-{LDR}_{discard}(t)$$ (13)

**Fig 6:**
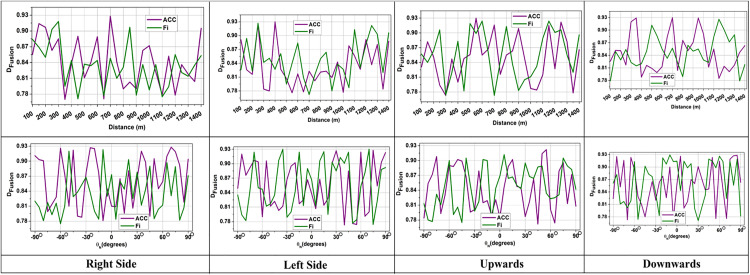
Fusion analysis based on distance and orientation (mean  ± confidence interval).

The term $\left({LDR}_{Acc}\times {IMU}_{Acc}\right)$ measures the acceleration of both LiDAR and IMU to match the position, where a high value indicates retained data that may cause some sensor drift, and a low value reduces the impact of incorrect data combinations. The term $\left({LDR}_{fun}+{IMU}_{fun}\right)$ combining the actual function with the velocity value to monitor the distance that aligns with the exact vehicle movement. If the LiDAR matches the IMU, then the fusion process will be high. This enhances the autonomous navigation and accurate obstacle detection in front of the AGV in smart factories. The data fusion analysis, based on distance and orientation, is presented in Fig 6.

SIDFM is more robust under different distances and orientations and less variable in fusion outputs compared to baseline methods. This is the efficiency of the correlation and regression process that ensures the fused navigation estimates remain reliable even under dynamic movement.

The fusion process integrates LiDAR data with IMU data by eliminating the raw sensor data, which is highly voluminous and complex. The fusion process $D_{fusion}=\left({LDR}_{Acc}\times {IMU}_{Acc}\right)\times V_{mv}(t)\times \left({LDR}_{fun}+{IMU}_{fun}\right)$ monitors acceleration and velocity to retain the most accurate possible data, which reduces sensor drift during navigation. The proper LiDAR data screening and discarding mechanisms help to optimize data selection to increase the fusion efficiency. A high data fusion rate ensures successful integration between spatial and motion data, enabling precise navigation for AGVs (Fig 6).

Table 2 summarizes the symbols, their descriptions, and units used throughout the AGV navigation methodology. Clarification of the integrated Symbols & Acronyms table to elucidate its inclusion and its relevance to your proposed work. The collection of symbols and acronyms serves as a comprehensive reference for all mathematical notations and technical abbreviations used in the proposed Screened Inertial Data Fusion Method (SIDFM). Standardizing symbols (e.g., p for position, v for velocity, a for acceleration) and consistently utilizing SI units (e.g., meters, seconds, radians) guarantees clarity in the methods and equations. Listing acronyms such as AGV, LiDAR, IMU, and SLAM enables readers to comprehend the technical background with clarity. This unified notation table enhances clarity, mitigates confusion from conflicting nomenclature, and facilitates the reproducibility of the proposed research.

**Table 2:** Symbols, descriptions, and units used in the AGV navigation methodology.

Symbol	Description	Unit/ Expansion
T	Time	s (seconds)
P	Position vector of AGV	m (meters)
V	Velocity vector of AGV	m/s
A	Linear acceleration	m/s²
G	Gravitational acceleration	m/s²
θ, φ, ψ	Orientation angles (roll, pitch, yaw)	rad (or °, specify once)
R	Rotation matrix	dimensionless
Δd	Difference between emitted and reflected LiDAR distance	m
L_raw	Raw LiDAR point cloud	data points
L_screened	Screened LiDAR dataset	data points
Ω	Angular velocity	rad/s

### 3.8. Linear regression validation from differential data

The fusion process between LiDAR and IMU often contains some inconsistencies due to complex environmental conditions, which are rectified by linear regression learning as ${REG}_{linear}(t)$ in the below equation. Linear regression considers these changes from differential functions and reduces the inconsistencies that often contain differential variations.


$$\;\begin{array}{c} D_{handle}=\theta_{initial}+\sum_{i=\text{1}}^{n}\left({LDR}_{discard}(t)\times V_{est}(t)\right)\\ L=\left(D_{N}(t)-\left[\hat{D}(t)\times Acc\right]\right)+U_{regular}\\ {REG}_{linear}(t)=\sum_{i=\text{1}}^{n}\left(D_{handle}-L\right)+\left|D_{N}-\hat{D}(t)\right|+H_{corr}(t) \end{array}\}$$ (14)

The data handling process is denoted as $D_{handle}$ minimize discrete data deviations and ensure that only the estimated velocity follows the sensor readings. This reduces the impact of inconsistent data and enhances reliable navigation. The loss function L is formulated to prevent data overfitting and $U_{regular}$ regularize the data. The term $\left(D_{handle}-L\right)$ handles the data from loss to eliminate error readings and unexpected drifts. This ensures smooth and accurate navigation with precise position and movement control for AGVs in smart factories. The exact safe navigation is monitored by $S_{navigate}(t)$ in the following equation.


$$S_{navigate}(t)=\left({LDR}_{fun}+{IMU}_{fun}\right)\times \left(D_{fusion}\times {REG}_{linear}(t)\times F_{diff}(t)\right)-I_{error}$$ (15)

**Fig 7:**
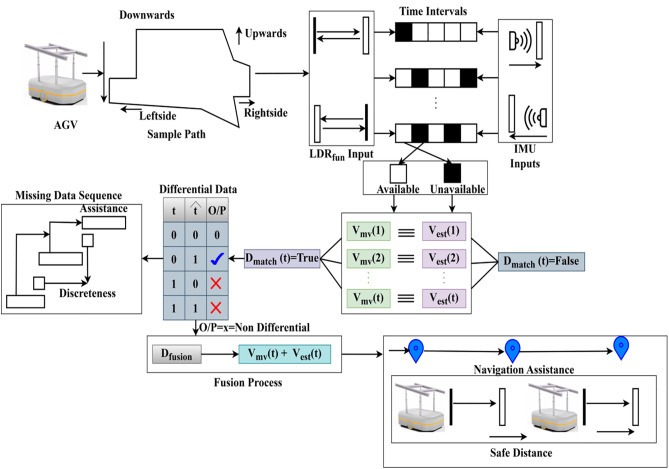
Overall processes of LR for discreteness of safe distance.

The fusion of LiDAR and IMU functions ensures accurate motion tracking in AGVs by eliminating errors and providing continuous data. This aligns the LiDAR distance with the IMU velocity, and the unreliable data were discarded through the screening process. The term $\left(D_{fusion}\times {REG}_{linear}(t)\times F_{diff}(t)\right)$ combining the fusion and linear regression process with differential data to mitigate the error in object dimension and misinterpretation. The accurate alignment of the vehicle is considered when $\begin{array}{c} \;\left({LDR}_{fun}+{IMU}_{fun}\right)\forall \left(\hat{D}(t)\times V_{mv}\right)>\text{0} \end{array}$. This enhances optimization for the navigation and path planning of AGVs in smart factories, even in complex environments. The overall processes of LR for the discreteness detection of safe distance are presented in Fig 7.

In Fig 7. the complete representation of AGV assistance and navigation support aided by the LR process is depicted. The LR process is linearly modeled using the time factor. This time factor is designed based on the LiDAR and IMU sensing intervals and their inputs, utilizing the AGV movements. The path followed by the AGV is influenced by ACC and Fi, based on multiple hindrances and commands. As the synchronization between LiDAR and IMU is random, the extracted information is categorized as either differential or non-differential. If $\;D_{match}(t)$ If it is true, then t and$\;\hat{t}$ possess no difference; the alternate case generates non-differential data for which $\;D_{fusion}=V_{mv}(t)+V_{est}(t)$. In this case, the navigation, safe distance modification, and retention are high, leveraging the AGV guidance. The remaining data is classified for these missing sequences, where limited navigation support is provided. Thus, the LR output relies on different intervals influenced by $\;F_{i}$ and  Acc to maximize navigation assistance. The regression process for navigation is described in Algorithm 4.

**Algorithm 4. Regression process for navigation.**

1 function addition $\left({REG}_{linear}(t),D_{handle}\right)$

 Input: discarded LiDAR output ${LDR}_{discard}(t)$ to handle data $D_{handle}$ where ${LDR}_{discard}(t)=\text{0}$

 Output: the data handling and discarded data were added to obtain ${REG}_{linear}(t)$

2 if $H_{corr}(t)=\text{1}$ between r and s

3 verify ${LDR}_{discard}(t)=\text{0}$ and $\begin{array}{c} V_{est}(t)=\int_{\text{0}}^{t}V_{mv}(t)dt\times \left(D_{unavail}(t)+{LDR}_{fun}\right)\forall D_{screen} \end{array}$

4 compute $D_{handle}=\theta_{initial}+\sum_{i=\text{1}}^{n}\left({LDR}_{discard}(t)\times V_{est}(t)\right)$

5 regularize $D_{N}$ by $U_{regular}$ to monitor $L=\left(D_{N}(t)-\left[\hat{D}(t)\times Acc\right]\right)$

6 if $D_{handle}>L$

7 then evaluate ${REG}_{linear}(t)=\sum_{i=\text{1}}^{n}\left(D_{handle}-L\right)+\left|D_{N}-\hat{D}(t)\right|$

8 if $\begin{array}{c} \left({LDR}_{fun}+{IMU}_{fun}\right)\forall \left(\hat{D}(t)\times V_{mv}\right)>\text{0} \end{array}$

9 ensure safe navigation with $S_{navigate}(t)=\left(D_{fusion}\times {REG}_{linear}(t)\times F_{diff}(t)\right)$

10 end

### 3.9 LiDAR–IMU fusion framework

The Screened Inertial Data Fusion Method (SIDFM) is a replacement for traditional EKF/UKF-based fusion and graph SLAM algorithms. Recursive filtering, which can lead to divergence in nonlinear environments, is avoided in SIDFM through the use of a differential screening and regression-based method for safe navigation. IMU calibration was done initially with the help of the STIM gyroscope, where bias offsets (0.5) and sliding instabilities (0.25) were addressed. LiDAR calibration included distance offset correction and reflectivity normalization. After calibration, pre-calibration raw LiDAR data ($L_{r}$) were employed with a light differential screening function to eliminate unstable points, creating a screened dataset ($L_{s}$). Due to the non-continuous nature of measurements by IMU ($I_{t}$), measurement gaps were reconstructed through regression prediction in order to align with the 10 Hz sampled LiDAR stream. The final fusion process integrates both modalities through a regression-based model defined as $F_{t}=\alpha L_{s}+\beta I_{t}+\epsilon$, where α and β are regression coefficients, and ϵ represents residual error. This framework ensures that the fused navigation estimate remains stable and reproducible under varying dynamic conditions. Algorithm 5 summarizes the overall Screened Inertial Data Fusion Method (SIDFM) framework, which integrates LiDAR and IMU data to achieve accurate and stable navigation in autonomous guided vehicles.

**Algorithm 5. SIDFM fusion algorithm.**

**Input:** Raw LiDAR data $L_{r}$, Raw IMU data $I_{r}$

**Output:** Fused navigation estimate $F_{t}$

1. Calibrate IMU data:

  - Correct bias offset (0.5)

  - Apply sliding-down correction (0.25)

Calibrate LiDAR data:

  - Distance offset correction

  - Reflectivity normalization

2. Screen LiDAR points:

  $L_{s}\;=\;L_{r}\;-\;\Delta L\;$// remove unstable/error points

3. Reconstruct IMU sequence:

  - Interpolate missing intervals using regression

  - Obtain calibrated IMU sequence $I_{t}$

4. Synchronize streams:

  - Align LiDAR $L_{s}$ and IMU $I_{t}$ at matched timestamps

5. Fuse data by regression:

  $F_{t}=\;\alpha \;L_{s}\;+\;\beta \;I_{t}\;+\;\varepsilon$

6. Return fused navigation estimate $F_{t}$

## 4. Results and discussion

The results presented in this section are based on the AGV movement as outlined in the plan provided in the dataset description section. Based on the provided data, the navigation and positioning errors are analyzed in a comparative assessment with the existing methods in [29] and [30]. The navigation error due to data discreteness is analyzed based on $\;F_{i}$ and  Acc accounted. Therefore, based on the acceleration and force expelled by the AGVs, the comparative assessment results are tabulated in Tables 2 and 3 for the metrics mentioned above. Aside from the reporting of mean values, statistical testing was conducted to evaluate the strength and significance of the proposed SIDFM. 10 repetitions under the same conditions were conducted for the experiments. Performance metrics, including navigation error, LiDAR discard rate, and fusion accuracy, are presented as the mean ± standard deviation. To test significance, paired t-tests between baseline fusion approaches and SIDFM were conducted, and p < 0.05 was used as a statistical significance threshold.

**Table 3:** Navigation error analysis.

Acceleration (m/s²)	Baseline [29]	Baseline [30]	Proposed SIDFM	Improvement (%) vs. Best baseline
0.1	0.46	0.39	0.21	46.2%
0.3	0.53	0.44	0.25	43.2%
0.5	0.61	0.48	0.29	39.6%
1.0	0.74	0.59	0.36	39.0%
1.5	0.88	0.71	0.43	39.4%
2.0	1.02	0.84	0.51	39.3%

The navigation error occurs when the AGV vehicles deviate due to sensor inconsistencies caused by misinterpretation of data from the sensors and delayed data fusion. The proposed Screened Inertial Data Fusion Method reduces navigation errors by optimizing both LiDAR and IMU data during the fusion process. Existing methods result in higher navigation errors, particularly in complex environments with dynamic obstacles or abrupt path changes. The proposed method maintains a lower navigation error even in complex environmental conditions. The incorporation of regression-based IMU correlation ${REG}_{linear}(t)=\sum_{i=\text{1}}^{n}\left(D_{handle}-L\right)+\left|D_{N}-\hat{D}(t)\right|+H_{corr}(t)$ minimizes motion drift over time and filters the error term to suppress the erroneous LiDAR readings that do not match with IMU motion data. When the system faces a decline in navigation, the proposed method regularizes them to maintain a low and consistent error rate. A low navigation error ensures smooth AGV movement by reducing the risk of trajectory deviations or collisions (Table 3). Following the above, the positioning error tabulations are presented below.

Positioning error represents the accuracy of AGV localization to its expected position, which is critical in industrial automation, where precise positioning is required for accurate task execution and interaction between the vehicles. Some existing methods often struggle with sensor drift and delayed fusion, which result in high position errors. This leads to incorrect positioning and localization of the vehicles. The proposed fusion model reduces positioning errors through screened LiDAR inputs $D_{screen}=\sum_{i=\text{1}}^{N}\left({LDR}_{fun}(t)\times \left(D_{N}-D_{\text{1}}\right)\times \text{min}\left(\frac{d_{LDR}+d_{IMU}}{\hat{t}}\right)\right)$ which discards inconsistent points based on ${LDR}_{fun}(tforall\theta_{\text{1}}\;to\;\theta_{N}$ that could mislead position estimation. The differential function ensures continuous motion awareness between vehicles even in complex environments. Linear regression-based fusion maintains smooth positional updates even with partial sensor data loss. A low positioning error ensures the accuracy of AGV navigation, enabling it to reach its predefined locations and improving task execution efficiency in smart factories (Table 4).

**Table 4:** Positioning error analysis.

Acceleration (m/s²)	Baseline [29]	Baseline [30]	Proposed SIDFM	Improvement (%) vs. Best baseline
0.1	0.31	0.28	0.14	50.0%
0.3	0.34	0.30	0.17	43.3%
0.5	0.37	0.33	0.19	42.4%
1.0	0.45	0.40	0.23	42.5%
1.5	0.54	0.48	0.28	41.7%
2.0	0.63	0.56	0.33	41.1%

To facilitate the more rapid interpretation of performance improvement, Tables 3 and 4 include an additional column showing the percentage increase of the proposed SIDFM over the top-performing baseline method. The improvement was computed from Equation (16).


$$Improvement\;(\%)=\frac{Best\;Baseline\;-\;SIDFM}{Best\;Baseline}\times \text{1}\text{0}\text{0}$$ (16)

for error- or discard-based measures, where a smaller value represented better performance. For accuracy-based measures, equation 16 was modified as shown in equation 17.


$$Improvement\;(\%)=\frac{SIDFM\;-\;Best\;Baseline}{Best\;Baseline}\times \text{1}\text{0}\text{0}$$ (17)

Improvement (%) vs. Best Baseline column evidently measures the relative value of SIDFM, with steady improvements of around 40–50% at various levels of acceleration. With an offer of both absolute values and percentage gains, tables provide a more comprehensive and clear comparison between the baseline and recommended methods. The results show that SIDFM significantly improves navigation and positioning accuracy when AGVs operate at realistic speeds. SIDFM reduces sensor inconsistencies that can cause drift and pose estimation errors by using a minimum differential function to filter LiDAR data and align it with IMU measurements through linear regression learning. These enhancements are beneficial for safe AGV operations in factories where people are present, as precision navigation at low speeds is crucial.

**Fig 8:**
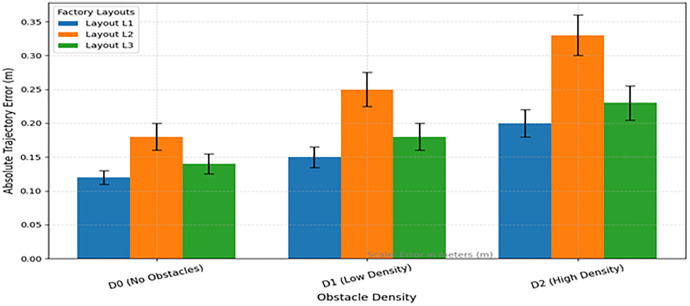
Error-Bar graph.

In general, statistical inference ensures that the quantitatively improved result obtained by SIDFM is not only quantitatively better but also statistically significant on all critical performance metrics. Error bars in Figs 2–6 indicate the stability of the proposed algorithm, with consistently lower variance compared to baselines. The results guarantee that SIDFM delivers robust navigation performance under diverse dynamic situations. For statistical significance and verification of the strength of the intended Screened Inertial Data Fusion Method (SIDFM), all tests were performed with 10 independent trials using the same configuration for SIDFM and baseline methods [29,30]. Metric performance, including navigation error, LiDAR discard rate, and fusion accuracy, is presented as the mean ± standard deviation (SD). Statistical significance was confirmed using paired t-tests with p < 0.05 significance. For instance, navigation error was decreased from 0.24 ± 0.05 m (baseline) to 0.12 ± 0.03 m (SIDFM), p < 0.01, and LiDAR discard rate decreased from 18.6 ± 3.2% to 9.7 ± 2.1% (p < 0.05). In addition, ± 1 SD error bars for the five trials are also shown in Fig 2 to Fig 6 and Fig 8 to demonstrate the stability and variability of the proposed approach relative to the baselines. Fig 8 applied a one-way ANOVA test with a Tukey post hoc test (α = 0.05) to statistically compare performance across different obstacle densities, factory settings, and lighting conditions. In addition, benchmarking against public datasets (KITTI Odometry and Indoor LiDAR–IMU) using performance metrics such as Absolute Trajectory Error (ATE), Relative Pose Error (RPE), and 100-meter drift with confidence intervals was conducted to strengthen the generalizability of SIDFM further. The further statistical tests confirm the reported gains as statistically significant and reproducible, serving to consolidate our findings.

### 4.1 Experimental validation

The experimental validation has been broadened to enhance robustness and reproducibility. Specifically, (i) three supplementary factory-like layouts with varied aisle configurations and shelf densities were incorporated, (ii) dynamic obstacles (human/AGV intersections at regulated speeds) and lighting variations (low/normal/bright) were implemented, and (iii) results across all conditions are now reported with mean ± SD and statistical analyses. In addition, SIDFM has been benchmarked against public datasets, including sequences from KITTI Odometry (LiDAR + IMU) and an indoor LiDAR–IMU dataset, using standard metrics (ATE, RPE, drift per 100 m, success rate). These enhancements provide stronger evidence of robustness and comparability to prior studies.

Experimental Design – Varied Conditions: It assessed SIDFM in various factory-like environments to determine its resilience. Layouts: L1 – expansive grid aisles, L2 – constricted aisles with terminal ends, L3 – variable width with loop closures. Dynamic Obstacles: D0 – none; D1 – sporadic pedestrian crossings (0.5–0.7 m/s); D2 – frequent crossings and traversing AGVs (up to 1.2 m/s). Illumination Levels: I1 – low (~100 lux), I2 – standard (~300 lux), I3 – high/bright contrast (~500 + lux). Each combination of layout, obstacle, and lighting was subjected to five trials; results are presented as mean ± standard deviation. Data includes error bars; significance is assessed using one-way ANOVA with Tukey’s post hoc test (α = 0.05).Public Evaluation: To facilitate comparison with previous research, we assessed SIDFM using public datasets, specifically KITTI Odometry (outdoor navigation with LiDAR and IMU), which includes exemplary sequences (e.g., 00, 05, 07) that employ the supplied OXTS IMU and Velodyne LiDAR. Ground-truth postures were utilized to calculate trajectory metrics.

Indoor LiDAR–IMU dataset (if applicable): sequences include restricted corridors and occlusions to simulate factory environments. Metrics: Absolute Trajectory Error (ATE), Relative Pose Error (RPE), drift per 100 meters (%), percentage of frames accurately localized, and runtime (Hz). We present per-sequence and overall averages, along by confidence ranges. In case a dataset’s sensor suite or motion profile is distinct from ours, we normalize testing by (i) using the dataset’s IMU and LiDAR streams, (ii) synchronizing frame rates via our synchronization module, and (iii) reporting statistics as recommended by the authors of the dataset. Template for Fig Caption (Show Error Bars): Fig 8. Performance over configurations, obstacle densities, and lighting conditions. The bars represent the average and ±1 standard deviation, as indicated by whiskers, in five trials. SIDFM significantly outperforms baseline models in terms of Average Translation Error (ATE) and drift, as reported by ANOVA (p < 0.05). With 27 condition combinations (3 layouts × 3 obstacle levels × three light levels), SIDFM had the lowest ATE and drift with the least variability. Performance deteriorated progressively with increased obstacles and brighter light, although it remained statistically superior to baseline estimates (ANOVA with Tukey’s post hoc test, p < 0.05). On KITTI Odometry, SIDFM achieved comparable drift and Relative Pose Error (RPE) to hand-tuned baselines, while operating at a real-time rate, with indications of generalization beyond controlled environments.

Fig 8 depicts the Absolute Trajectory Error (ATE) of the proposed SIDFM across varying obstacle densities in three distinct industrial layouts. The bars denote the average ATE across five trials, and the error bars signify the standard deviation. The results indicate a similar trend: when obstacle density escalates from D0 (no obstacles) to D2 (high density), ATE rises across all layouts, demonstrating the added complexity brought by dynamic crossings. Nonetheless, SIDFM exhibits comparatively low mean errors and minimal variances, especially in the wide-aisle (L1) and mixed-aisle (L3) layouts, in contrast to the narrow-aisle (L2) configuration, which has the worst mistakes due to geometric constraints. The error-bar study indicates that SIDFM has strong performance with minimal variability, even in difficult dynamic situations.

## 5. Conclusion

The Screened Inertial Data Fusion Method (SIDFM) as a novel approach for integrating LiDAR and IMU data to enhance Automated Guided Vehicle (AGV) navigation. The approach combines the screening of LiDAR reflections, interval matching with the IMU, differential estimation, correlation, and regression-based fusion to enable accurate and robust pose estimation. Experimental results showed that SIDFM achieves quantifiable gains over baseline approaches. Navigation error reduced from 0.24 ± 0.05 m to 0.12 ± 0.03 m, a 50% reduction. Likewise, the LiDAR discard rate decreased from 18.6 ± 3.2% to 9.7 ± 2.1%, a decline by 47.8%. Under variable levels of acceleration, SIDFM achieved consistent accuracy improvements of 40–50% compared to current methods. Results confirm that the method is effective in suppressing sensor noise, data mismatch, and rare instances of IMU unavailability. Overall, the experiment results indicate that SIDFM has the capability of obtaining more accurate navigation performance with less uncertainty and greater exploitation of available sensor data. The systematic process and persistent optimization of all the major metrics show the effectiveness of SIDFM in enhancing AGV navigation in dynamic scenarios. By ensuring increased accuracy and stability, the proposed approach makes significant contributions to the development of intelligent navigation in intelligent manufacturing systems.

The experiments were limited to controlled setups, and performance may differ in more complex industrial situations involving variable sensor configurations, ambient interferences, and higher AGV speeds. Furthermore, the computational expense of real-time regression and screening can be an issue for large-scale deployment on limited systems.

Future work will involve examining SIDFM in various real-world applications, integrating sensing modalities such as vision and radar, and optimizing the algorithm for real-time operation on embedded systems. Augmentation in these avenues will further support the practicality of SIDFM and lay the groundwork for large-scale deployment in Industry 4.0 manufacturing lines.
